# Regulating heat conduction of complex networks by distributed nodes masses

**DOI:** 10.1038/s41598-021-85011-0

**Published:** 2021-03-09

**Authors:** Kezhao Xiong, Zhengxin Yan, You Xie, Zonghua Liu

**Affiliations:** 1 College of Science, Xi’an University of Science and Technology, Xi’an, 710054 People’s Republic of China; 2 Department of Physics, Fudan University, Shanghai, 200433 People’s Republic of China; 3 School of Physics and Electronic Science, East China Normal University, Shanghai, 200062 People’s Republic of China

**Keywords:** Statistical physics, thermodynamics and nonlinear dynamics, Complex networks, Statistical physics, Thermodynamics

## Abstract

Developing efficient strategy to regulate heat conduction is a challenging problem, with potential implication in the field of thermal materials. We here focus on a potential thermal material, i.e. complex networks of nanowires and nanotubes, and propose a model where the mass of each node is assigned proportional to its degree with $m_i\sim k_i^{\alpha }$, to investigate how distributed nodes masses can impact the heat flow in a network. We find that the heat conduction of complex network can be either increased or decreased, depending on the controlling parameter $\alpha$. Especially, there is an optimal heat conduction at $\alpha =1$ and it is independent of network topologies. Moreover, we find that the temperature distribution within a complex network is also strongly influenced by the controlling parameter $\alpha$. A brief theoretical analysis is provided to explain these results. These findings may open up appealing applications in the cases of demanding either increasing or decreasing heat conduction, and our approach of regulating heat conduction by distributed nodes masses may be also valuable to the challenge of controlling waste heat dissipation in highly integrated and miniaturized modern devices.

## Introduction

Fourier’s law $J=-\kappa \nabla T$ is the fundamental principle of heat conduction, where $\kappa$ is the heat conduction coefficient that in the past was considered as an inherent property of a system, independent from the shape and size. However, Lepri et al. investigated the thermal transport in microsystems utilizing the FPU model, and found that in a one-dimensional system $\kappa$ can diverge with the increase of the system size with conservation of momentum^1,2^. This drew great attention in the field of heat conduction in nano-scale systems, and has then been confirmed by extensive research, for instance numerical simulations, theoretical analyses, and experimental investigations on quasi-one-dimensional systems, such as the graphene, nanowires and nanotubes^3–12^ etc. Moreover, the horizon of researches along this line were recently extended to thermal rectification^13–15^, negative differential thermal resistance^16,17^, and the effect of thermal-siphon^18^ etc.

Nanotubes and nanowires have excellent thermal, electrical, mechanical, optical and chemical properties^19,20^. However, it is still extremely difficult to reliably control the growth and arrangement of single nanotubes and nanowries in industrial production, as a result, devices comprises single nanotubes and nanowires are still not widely used in reality. A strategy to sidestep this difficulty is use networks of nanotubes or nanowires, instead of just single nanotubes and nanowires. Recently, it is reported that these network structure of nanotube and nanowire can serve as new nanomaterials for thermal management, and can be potentially applied in large-scale transparent conductors, solar cells, field effect transistors, sensors, flat panel displays and interface devices for living cells^21–23^ etc. Therefore, it becomes crucial to develop models for quasi-nano network materials, to explore its thermal transport properties, and to investigate the mechanisms underlying these properties.

**Figure 1:**
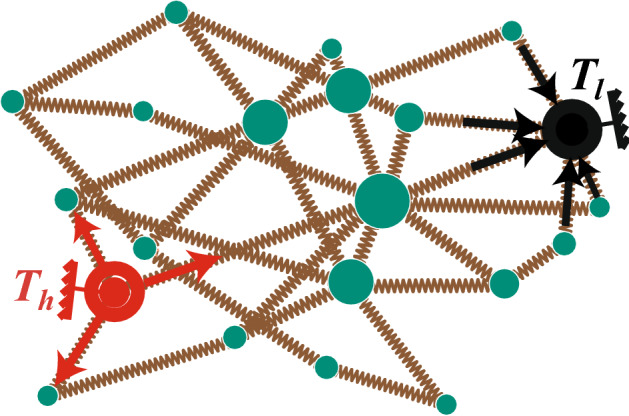
Schematic representation of the network model with distributed nodes masses, where the masses of nodes are correlated to the degrees of nodes by Eq. (1) with $\alpha =1.5$, and the nodes with red and black circles are selected as the heat source nodes contacting two thermostats with high temperature $T_{h}$ and low temperature $T_{l}$, respectively. The arrows denote the directions of heat fluxes.

Along the line of heat conduction in networks, some important progresses have been achieved. Liu et al. firstly studied the heat conduction on coupled chains and found that coupling will cause an interface thermal resistance^24^. Then, they constructed the first model of heat conduction on complex networks and found that both the degree distribution and clustering coefficient will seriously influence the network thermal transport^25^. Volkov et al. studied the scaling laws of thermal conductivity in random networks of straight conducting nanofibers and found that the heat transport can be strongly enhanced by the self-organization of carbon nanotubes^26^. Xiong et al. further revealed two abnormal effects of heat conduction, i.e. thermal rectification and thermal siphon in physical network model^18,27,28^. However, despite its broad implication, little is known on how to regulate heat conduction in complex networks. In fact, this problem is important in many cases where either increasing or decreasing of heat conduction may be demanded. Increase of heat conduction is essential for eliminating waste during heat transport^29^. In some other scenarios, such as thermal insulation, decrease or preventing of heat conduction becomes the key element, which has been a hot topic in the filed of thermal energy storage^30,31^. These all lead to the demand of exploring the mechanism that regulates heat conduction in complex networks.

Motivated by this problem, we investigate a network system with nonidentical node mass, with the aim to understand how the mass distribution can impact the pattern of heat conduction. This idea of distributed nodes masses is applicable in practical situations. Actually, some works have shown that in low-dimensional systems, changes of nodes masses can significantly influence the properties of thermal transport^32–34^. However, these works are based on regular lattice topology but not networks, and the mass distributed to each node is also independent from the network property, such as node degree. As far as I know, nanonetworks are formed by welding the intersections of nanowires or nanotubes, and the intersections after welding are usually called nanojunctions^35,36^. During the welding process, a large number of atoms are deposited at the nanojunctions, thereby increasing the mass of the nanojunctions, and generally the size of the nanojunction increases with increase of the number of intersecting nanowires or nanotubes at nanojunction^37–39^. In complex networks, nanojunctions are analogous to network nodes, and the number of nanowires crossing at nanojunctions can be regarded as nodes degree. To counter this, we construct a model, in which each node is assigned a mass that is dependent on its degree, i.e. $m_i\sim k_i^{\alpha }$, and focus on how this correlation and network parameters affect network thermal transport. We find that the heat flux on network can be either increased or decreased, depending on the controlling parameter $\alpha$. Further, we find that there is an an optimal value of $\alpha =1$ independent of network topologies, and at which the heat conduction is most benefited. Moreover, we find that the temperature distribution on complex network is also seriously influenced by the controlling parameter $\alpha$. A brief theoretical analysis is provided to explain these results.

## Results

**The network model with distributed nodes masses.** We construct the network model with distributed nodes masses by followings. Firstly, we use the method proposed in Ref.^40^ to construct a complex network, see the schematic figure of Fig. 1. In details, we start from *q* fully connected nodes, and then at each time step a node with *g* edges is added to the network and connects to a existing node *i* with the probability of $\Pi _i \sim (1-p)k_i+p$, where $k_{i}$ is the degree of the existing node i, and $0 \le p \le 1$ is a control parameter. Iterating this procedure, we general a network with *N* nodes and average degree $\langle k\rangle =2g$. For the case of $p=0$, the nodes added in the system at each step tend to connect to the nodes with large degrees in the original network, thus forming a scale-free network with degree distribution yielded to power-law distribution. For the case of $p=1$, the new edges at each step will randomly connect to the existing nodes, thus forming a random network with degree distribution satisfying exponential distribution. While for the case of $0<p <1$, it generates a complex network between the scale-free and random networks. If there is no special statement, we set $q=3$ and $g=2$, i.e. $\langle k\rangle =4$ in this work.

Then, in the second step, we set the mass of node *i* as

1 $$\begin{aligned} m_i=\frac{k_i^{\alpha }}{M}, \end{aligned}$$

where $k_{i}$ is the degree of node *i* and $M=\sum _{i=1}^{N}m_i$. Here, we have normalized the total mass of network to eliminate its influence on the results. For the case of $\alpha =0$, the mass of each node will be the same in the network, i.e. $m_i=1/N$. While for the case of $\alpha>0$, the mass of node will increase nonlinearly with the increase of node degree. Especially, when $\alpha$ is large enough, the mass of entire network will be concentrated to the nodes with larger degrees, and the mass of other nodes will be close to 0. This is unrealistic, thus we limit the maximum value of $\alpha$ to 3. Figure 1 shows an example for the case of $\alpha =1.5$, where the node mass is reflected by the node size.

After these two steps, we randomly choose two nodes from the network as the source nodes and let them contact a high- and low-temperature Langevan thermostat^3,41^, respectively. The two source nodes obey fixed boundary condition, as shown in Fig. 1.

Without loss of generality and simplicity, the Hamiltonian of the network can be defined as

2 $$\begin{aligned} H=\sum _{i=1}^N\left[ \frac{p^{2}_{i}}{2m_{i}}+V_{i}\left( x_{i}\right) \right] , \end{aligned}$$

and the potential

3 $$\begin{aligned} V_{i}\left( x_{i}\right) =\frac{1}{2}\sum _{j=1}^{k_{i}}\left[ \frac{1}{2}\left( x_{i}-x_j\right) ^{2}\right] , \end{aligned}$$

where *i* runs through all the nodes of the network, $x_{i}$ represents the displacement from the equilibrium position of the *i*-th node. The dynamics of the two source nodes satisfy

4 $$\begin{aligned} \frac{d{p}_{h}}{d t}= & {} -\frac{\partial H}{\partial x_{h}}+\Gamma _{h}-\gamma {p}_{h}, \end{aligned}$$

5 $$\begin{aligned} \frac{d{p}_{l}}{d t}= & {} -\frac{\partial H}{\partial x_{l}}+\Gamma _{l}-\gamma {p}_{l}, \end{aligned}$$

where $\Gamma _{h,l}$ are the Gaussian white noises with

6 $$\begin{aligned}&\langle \Gamma _{h,l}(t)\rangle =0, \end{aligned}$$

7 $$\begin{aligned}&\langle \Gamma _{h}(t)\Gamma _{h}(0)\rangle =2\gamma k_{B}m_{h}T_{h}\delta (t), \end{aligned}$$

8 $$\begin{aligned}&\langle \Gamma _{l}(t)\Gamma _{l}(0)\rangle =2\gamma k_{B}m_{l}T_{l}\delta (t), \end{aligned}$$

where $m_{h}(m_{l})$ is the mass of source node, $T_{h}(T_{l})$ is the temperature of source node, $k_{B}$ is the Boltzmann constant, and we adopt the dimensionless unit by setting $k_{B}=1$. We set the friction coefficient $\gamma =5$ in this work, which is within the recommended range of $\gamma \in (1,100)$^41^ so that a meaningful physics can be obtained. Besides the two source nodes, the motion of other nodes in the network obeys the canonical equation

9 $$\begin{aligned} \frac{d{p}_{i}}{d t}=-\frac{\partial H}{\partial x_{i}}. \end{aligned}$$

After transient process, the thermal transport on network will reach a steady state. The local temperature of node *i* can be defined as^3^

10 $$\begin{aligned} T(i)=\langle p^{2}_{i}/m_{i} \rangle , \end{aligned}$$

and the heat flux on the edge between two adjacent nodes *i* and *j* can be calculated by the formula^3,32^

11 $$\begin{aligned} J_{ij}=\langle \dot{x}_{i}\partial V/\partial x_{j}\rangle , \end{aligned}$$

where $\langle \cdots \rangle$ is the long time average.

### Numerical simulations

In numerical simulations, we set the network size $N=300$, average degree $\langle k\rangle =4$, and temperature of the high and low thermostats as $T_h=0.9$ and $T_l=0.1$ respectively, if without specific illustration. We randomly choose two nodes as the source nodes to contact the high and low thermostats, respectively. Let *J* be the total heat flux on the network, defined as the sum of the heat flows from the high-temperature source node to all its neighbors, or the sum of the heat flows from the network to the low-temperature source node, see the arrows in Fig. 1. After transient process, *J* will arrive a constant which can measure the efficiency of thermal transport in the network^3^.

**Figure 2:**
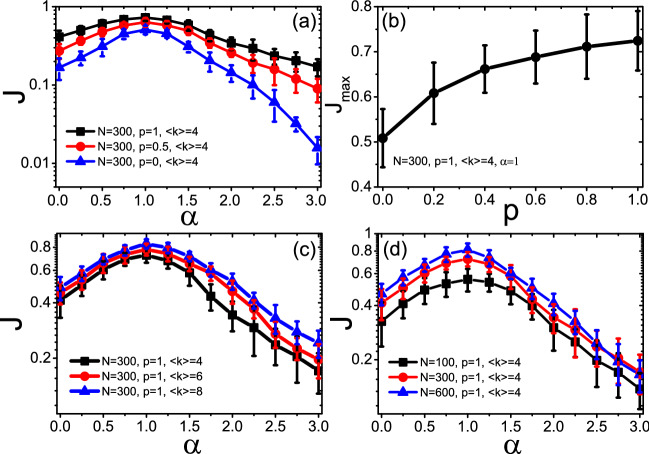
Influence of the controlling parameter $\alpha$ and network structure on the total flux *J*, with averaging over 50 realizations and randomly chosen source nodes. (**a**) *J* versus $\alpha$ for $N=300$, $\langle k\rangle =4$ and different *p*, where the “squares”, “circles” and “triangles” represent the results for the networks with $p=1, 0.5$ and 0, respectively. (**b**) Dependence of the maximum flux $J_{max}$ on the parameter *p* for $\alpha =1$. (**c**) *J* versus $\alpha$ for $N=300$, $p=1$ for different $\langle k\rangle$, where the “squares”, “circles” and “triangles’ represent the results for $\langle k\rangle =4, 6$ and 8, respectively. (**d**) *J* versus $\alpha$ for $p=1$, $\langle k\rangle =4$ for different *N*, where the “squares”, “circles” and “triangles” represent the results for $N=100, 300$ and 600, respectively.

In order to explore the influence of distributed nodes masses on thermal transport, we make extensive numerical simulations on thermal transport for the networks with different parameter $\alpha$, degree distribution, network size and average degree etc. Figure 2a shows the dependence of the total heat flux *J* on the parameter $\alpha$ for different *p*, where the “squares”, “circles” and “triangles” represent the results for the networks with $p=1, 0.5$ and 0, respectively. All the results are averaged over 50 realizations with randomly chosen source nodes. We find that all the values of *J* increase before $\alpha <1$ and reach the maximum $J_{max}$ at $\alpha =1$, then decrease with the further increase after $\alpha >1$. When $\alpha$ is far away from unity, *J* quickly decreases across roughly two orders of magnitude till $\alpha =3$.

Figure 2b shows the dependence of the maximum flux $J_{max}$ on the parameter *p* for $\alpha =1$. We see that $J_{max}$ increases monotonically with *p*, confirming that the random network benefits heat conduction better than the scale-free network. Figure 2c shows the influence of average degree $\langle k\rangle$ on *J* for $N=300$ and $p=1$ where the “squares”, “circles” and “triangles” represent the results for $\langle k\rangle =4, 6$ and 8, respectively. We see that the curve of $\langle k\rangle =8$ is higher than that of $\langle k\rangle =6$ and then both are higher than that of $\langle k\rangle =4$, indicating that larger $\langle k\rangle$ is beneficial for heat conduction. Figure 2d shows the influence of network size *N* on *J* for $\langle k\rangle =4$ and $p=1$ where the “squares”, “circles” and “triangles” represent the results for $N=100, 300$ and 600, respectively. We see that the curve of $N=600$ is higher than that of $N=300$ and then both are higher than that of $N=100$, implying that larger *N* favorites heat conduction.

**Figure 3:**
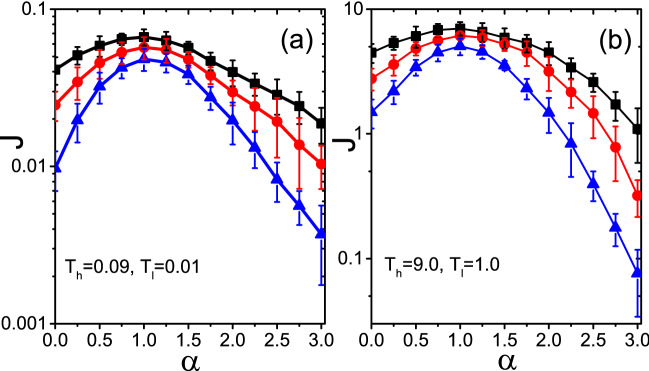
Influence of the controlling parameter $\alpha$ and the temperature of thermostats on the total flux *J*, with averaging over 50 realizations and randomly chosen source nodes. (**a**) For the case of $T_h=0.09$ and $T_l=0.01$, *J* versus $\alpha$ for $N=300$, $\langle k\rangle =4$ and different *p*, where the “squares”, “circles” and “triangles” represent the results for the networks with $p=1, 0.5$ and 0, respectively. (**b**) For the case of $T_h=9.0$ and $T_l=1.0$, *J* versus $\alpha$ for $N=300$, $\langle k\rangle =4$ and different *p*, where the “squares”, “circles” and “triangles” represent the results for the networks with $p=1, 0.5$ and 0, respectively.

Notably, from the Fig. 2a,c,d that all the *J* reach their maximum value $J_{max}$ at $\alpha =1$, indicating that a mass distribution with $\alpha =1$ in Eq. (1) can optimize the heat conduction. In order to further explor the influence of thermostats’ temperature on this characteristic, we further research the dependence of the total heat flux *J* on the parameter $\alpha$ for the cases of $T_h(T_l)=0.09(0.01)$ and $T_h(T_l)=9.0(1.0)$, respectively. From the results in Fig. 3a,b, we can confirm that the temperature of thermostats can not affect the conclusion that $\alpha =1$ is beneficial for heat conduction. We will discuss its physical mechanism later.

**Figure 4:**
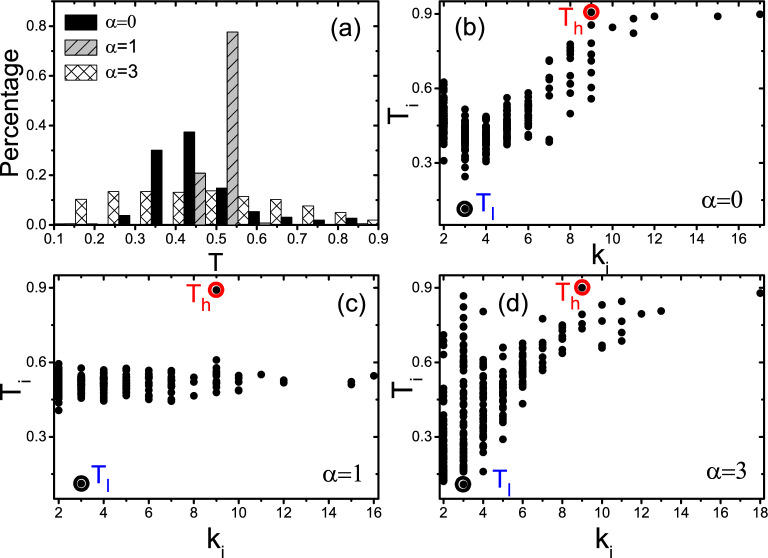
Influence of distributed nodes masses on the distribution of nodes temperatures for $N=300$, $p=1$ and $\langle k\rangle =4$, where the circles with $T_h$ and $T_l$ are the two source nodes with high and low temperatures, respectively. (**a**) Represents the distributions of nodes temperatures where the three kinds of columns represent the cases for $\alpha =0, 1$ and 3, respectively. (**b**–**d**) represent the dependence of node temperature $T_i$ on its degree $k_i$ for $\alpha =0, 1$ and 3, respectively.

On the other hand, we find that the controlling parameter $\alpha$ can also seriously influence the distribution of nodes temperatures. Take the random network of $p=1$ as an example. The three kinds of columns of Fig. 4a show the distributions of nodes temperatures for $\alpha =0, 1$ and 3, respectively. It follows an approximate Poisson distribution for the case of $\alpha =0$, a narrow range with a high peak for the case of $\alpha =1$, and a uniform distribution for the case of $\alpha =3$. Therefore, the temperature distribution for different $\alpha$ can be distinct from each other. We further confirm that these significant differences are consistent for other network with different parameters of *p*, *N* and $\langle k\rangle$. To understand the mechanism of these significant differences, we show in Fig. 4b–d the dependence of node temperature $T_i$ on its degree $k_i$ for $\alpha =0, 1$ and 3, respectively. Node temperature $T_i$ depends strongly on $k_i$ for $\alpha =0$ (Fig. 4b) and $\alpha =3$ (Fig. 4d). However, it is independent of $k_i$ for $\alpha =1$ (Fig. 4c), indicating a temperature platform is formed in the case of $\alpha =1$. This is an interesting phenomenon and can help us to understand the mechanism of the observed maximum $J_{max}$ at $\alpha =1$. In general, there are two factors influencing the heat conduction of node *i*. One is the input and output connections, i.e. more input and output connections, more heat conduction. As the numbers of input and output connections are proportional to $k_i$, the heat conduction will be also proportional to $k_i$. Another is the mass of node *i*. As a larger mass reduces the heat conduction (see Eq. (2)), the case of $\alpha =1$ indicates a inverse proportion to $m_i$, i.e. $k_i$. These two factors compete and reach a balance at all the nodes and thus make a uniform heat transport on the network. While for the cases of either $\alpha >1$ or $\alpha <1$, the balance is broken. The consequence is that heat transport is significantly reduced at those nodes with either larger $k_i$ (for the case of $\alpha >1$) or smaller $k_i$ (for the case of $\alpha <1$), thus reduce the total flux *J* of network. This competition and balance leads to the optimization of $\alpha =1$ for heat conduction, i.e., $J_{max}$ at $\alpha =1$.

**Figure 5:**
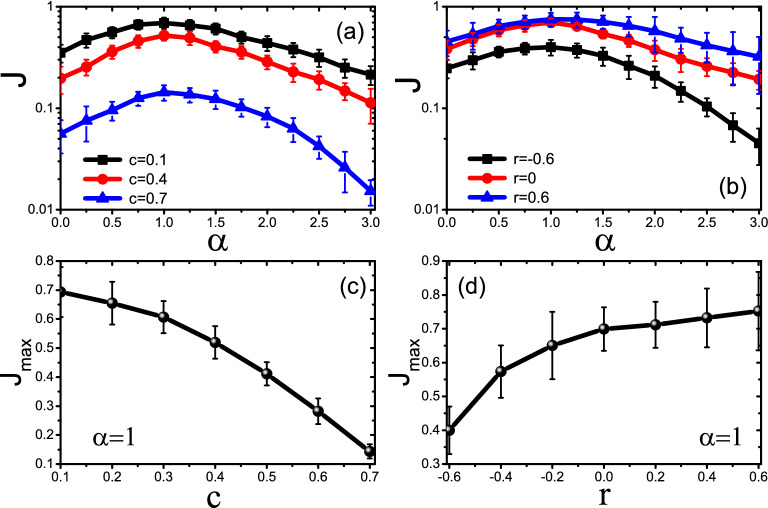
Influence of the clustering coefficient *c* and assortativity coefficient *r* on the total heat flux *J* for $N=300$ and $\langle k\rangle =4$, with averaging over 50 realizations and randomly chosen source nodes. (**a**) represents the influence of *c* on *J* where the “squares”, “circles” and “triangles” represent the cases of $c = 0.1, 0.4$ and 0.6, respectively. (**b**) represents the influence of *r* on *J* where the “squares”, “circles” and “triangles” represent the cases of $r=-0.6, 0$ and 0.6, respectively. (**c**) Dependence of $J_{max}$ on *c* for $\alpha =1$. (**d**) Dependence of $J_{max}$ on *r* for $\alpha =1$.

Further, we study how the other parameters of network influence heat conduction, such as the clustering coefficient *c* and assortativity coefficient *r*, see Methods for details. For convenience of discussion, we choose the random network of $p=1$. Figure 5a shows the influence of *c* on *J* where the three curves represent the cases of $c = 0.1, 0.4$ and 0.7, respectively. We see that all the three curves are bell-shaped and the maximum $J_{max}$ is still located at $\alpha =1$, i.e. consistent as in Fig. 2a,c,d. Similarly, Fig. 5b shows the influence of *r* on *J* where the three curves represent the cases of $r=-0.6, 0$ and 0.6, respectively. We see that the maximum $J_{max}$ is also obtained at $\alpha =1$. Figure 5c shows the dependence of $J_{max}$ on *c* for $\alpha =1$. We see that $J_{max}$ decreases with the increase of *c*, which is consistent with Ref.^25^. Figure 5d shows the dependence of $J_{max}$ on *r* for $\alpha =1$. We see that $J_{max}$ increases with *r*, indicating that a strong assortativity coefficient *r* favorites heat conduction. Combining results in Fig. 5, network characters such clustering coefficient and assortativity dose not impact the optimal $\alpha$, indicating that $J_{max}$ at $\alpha =1$ is a robust feature for heat conduction in networks.

**Figure 6:**
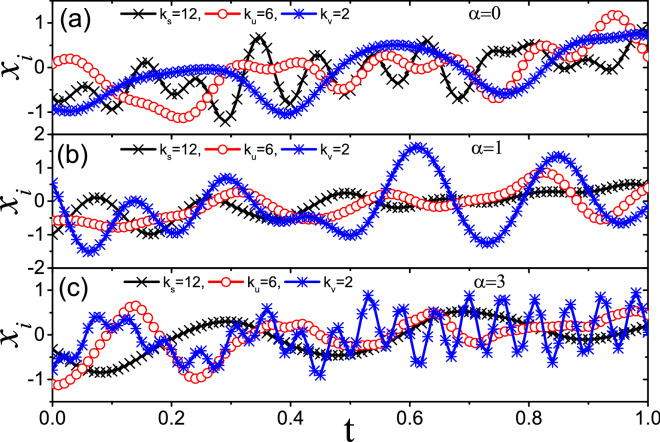
Time series of arbitrarily chosen nodes *s*, *u* and *v* from the random network of $p=1$, where the degrees of the nodes *s*, *u* and *v* are 12, 6 and 2, respectively. (**a**–**c**) represent the cases of $\alpha =0, 1$ and 3, respectively.

### Mechanism of the maximum total flux J of networks

To go deeper to the mechanism of the maximum $J_{max}$ from $\alpha =1$, we analyze the time series of different nodes. We find that the oscillation frequencies are different from node to node and seriously depend on the parameter $\alpha$. To show it in details, we take the random network with $p=1$ and arbitrarily choose three nodes *s*, *u* and *v* as examples. Figure 6a shows their time series for the case of $\alpha =0$ where the degrees of the three nodes are $k_{s}=12, k_{u}=6$ and $k_{v}=2$, respectively. We see that the node *s* has the largest oscillation frequency while the node *v* has the smallest one, indicating that the frequencies are proportional to their degrees. Figure 6b shows their time series for the case of $\alpha =1$. We find that their frequencies are approximately the same, confirming that heat fluxes go freely from one node to another in the network. Figure 6c shows the results for the case of $\alpha =3$. We see that the node *s* has the smallest oscillation frequency while the node *v* has the largest one, in contrast to the case of Fig. 6a. In the next section, we will show that the match of oscillation frequency among nodes is the key factor to influence the heat conduction and thus results in the observations in Figs. 2, 3, 4 and 5.

**Figure 7:**
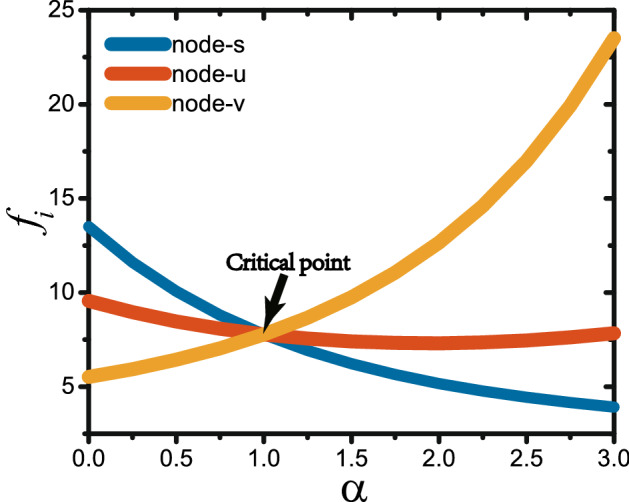
Dependence of the spectrum width $f_{i}$ on the control parameter $\alpha$ where the three curves represent the cases of nodes *s*, *u* and *v*, respectively.

### Theoretical analysis

As we all know, phonon is the carrier of heat conduction in vibration systems, thus we need to focus on the phonon spectrum of network nodes to explain the above numerical simulation results. It can be known from the Refs.^18,27^ that the frequency width $f_{i}$ of a node *i* in a complex network is related to its degree $k_{i}$

12 $$\begin{aligned} 0<f_i< \frac{1}{\pi }\sqrt{\frac{k_i}{2}}. \end{aligned}$$

**Figure 8:**
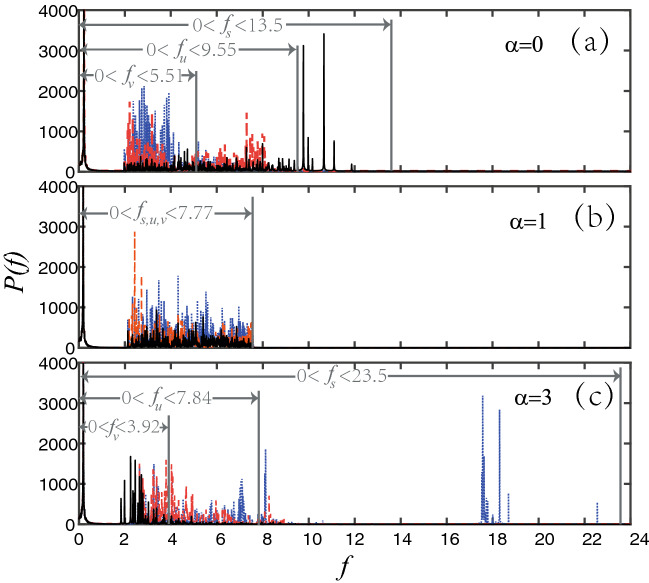
Power spectra of the time series in Fig. 6a–c where the black solid line, red dash line and blue dot line correspond to the nodes *s*, *u* and *v*, respectively. The width of grey arrows is the theoretical result by Eq. (13). The other parameters are the same as in Fig. 6.

What needs to be emphasized here is that Eq. (12) is only suitable for the case of uniform distribution, i.e. $\alpha =0$. Based on the derivation of Eq. (12), we can easily obtain the following Eq. (13) for other cases of non-uniform distribution in our new model

13 $$\begin{aligned} 0<f_i< \frac{1}{\pi }\sqrt{\frac{k_{i}^{1-\alpha }M}{2}}. \end{aligned}$$

From Eq. (13) the frequency width $f_{i}$ of node *i* is determined by its degree $k_{i}$ and the control parameter $\alpha$. Specifically, it will be independent of the node degree when $\alpha =1$. Figure 7 shows the results for the three nodes *s*, *u* and *v*. We see that the three curves intersect at $\alpha =1$. Moreover, with the increase of $\alpha$, the frequency width decreases monotonically for the node *s*, keeps approximate constant for the node *u*, but increases monotonically for the node *v*. These results are consistent with all the frequencies in Fig. 6. To further verify these theoretical results, we carry out the Fourier transform for the time series of Fig. 6. Figure 8a–c show the power spectra of time series, corresponding to Fig. 6a–c, respectively. They are completely consistent with the theoretical results from Eq. (13).

## Discussion

From the aspect of energy transport, we can also understand the optimal $\alpha =1$ for heat conduction. It is now well known that the carrier of heat flow is phonons. Whether the widths of phonon spectra of nodes are consistent with each other significantly affect the thermal transport efficiency^17^. According to the theoretical analysis and the power spectra of time series, we discover that the spectra widths are the same from node to node for $\alpha =1$ and thus result in the highest efficiency of energy transport, i.e. the maximum $J_{max}$.

The findings in this work may have potential applications. Eliminating waste heat is becoming more and more important in highly integrated and miniaturized devices and many new materials with excellent thermal conductivities are gradually discovered such as carbon nanotubes^42^, graphene^43^, and cubic boron arsenide^44^ etc. On the other hand, reducing or preventing heat conduction is also needed in other cases such as thermal insulation^30,31^. Thus, regulating heat conduction is the key for its applications. Regarding our model, the key point is how to implement the relationship of Eq. (1) in reality. For this purpose, we may borrow the idea of brain networks, where each node represents an area of brain and thus contains a number of regions of interest (ROIs)^45–49^. By this idea, we can also let each node of Eq. (1) be a community or subnetwork with dense connections. In this way, the mass of a node can be either large or small by easily changing its size of community. Thus, the approach of distributed nodes masses is qualified for the regulating of heat conduction in reality, i.e. implement the purpose of either good or poor heat conduction.

In conclusions, we propose a model of distributed nodes masses to study the regulating of heat conduction in complex networks. By this model, we can implement the purpose of controlling the total heat flux of network. The numerical results are confirmed by theoretical analysis. These findings may shed light on developing strategies of regulating heat conduction in systems with complex structure and suggest an appealing way to produce new thermal insulation materials.

## Methods

### The scheme of adjusting clustering coefficient *c* or assortativity coefficient *r*

For a complex network with fixed degree distribution, its topology can be still changed by adjusting its clustering coefficient *c* or assortativity coefficient *r*. Clustering coefficient describes the closeness between the adjacent nodes of a node in the network, which can be calculated as follows^50^:

14 $$\begin{aligned} c=\frac{1}{N}\sum _{i=1}^N \frac{E_i}{k_i(k_i-1)/2}, \end{aligned}$$

where $k_{i}$ is the degree of node *i*, $E_{i}$ is the number of edges between all neighbors of node *i*, and $0<c <1$. With the increase of *c*, the network become highly clustered.

The assortativity coefficient is another important quantity describing the preference of the connections between nodes in the complex network. Specifically, the network is defined as an assortativity network if a node tends to connect another node with similar degree; otherwise, the network is a disassortativity network if a node tends to connect another node with large degree difference. The assortativity coefficient *r* of network can be calculated as follows^51^:

15 $$\begin{aligned} r=\frac{\langle k_{i}k_{j}\rangle -\langle \left( k_{i}+k_{j}\right) /2\rangle ^{2}}{\langle \left( k^{2}_{i}+k^{2}_{j}\right) /2\rangle -\langle \left( k_{i}+k_{j}\right) /2\rangle ^{2}}, \end{aligned}$$

where $k_{i}$ and $k_{j}$ denote the degree of two endpoints on any connected edge in the network, and $\langle \cdot \rangle$ represents the average of all connected edges in the network. In this work, we use Kim’s reconnection method^52^ to adjust the clustering coefficient *c* and assortativity coefficient *r*. In details, we first randomly select two edges in the network. Suppose one is $A-B$ and another is $C-D$. Then, we disconnect $A-B$ and $C-D$ and reconnect them into $A-D$ and $B-C$. It should be emphasized that repeated edges must be prohibited in this process. The advantage of this method is that the degree of nodes is not changed in the process of reconnection, i.e. the degree distribution of network is maintained.
